# A Label-Free Carbohydrate-Based Electrochemical Sensor to Detect *Escherichia coli* Pathogenic Bacteria Using D-mannose on a Glassy Carbon Electrode

**DOI:** 10.3390/bios13060619

**Published:** 2023-06-05

**Authors:** Sakineh Hargol Zadeh, Soheila Kashanian, Maryam Nazari

**Affiliations:** 1 Faculty of Chemistry, Applied Chemistry Department, Razi University, Kermanshah 6714414971, Iran; 2 Nanobiotechnology Department, Faculty of Innovative Science and Technology, Razi University, Kermanshah 6714414971, Iran

**Keywords:** electrochemical sensing, adhesion FimH, electrochemical impedance spectroscopy, gold nanoparticles, electrodeposited

## Abstract

Controlling water and food contamination by pathogenic organisms requires quick, simple, and low-cost methods. Using the affinity between mannose and type I fimbriae in the cell wall of *Escherichia coli* (*E. coli*) bacteria as evaluation elements compared to the conventional plate counting technique enables a reliable sensing platform for the detection of bacteria. In this study, a simple new sensor was developed based on electrochemical impedance spectroscopy (EIS) for rapid and sensitive detection of *E. coli*. The biorecogniton layer of the sensor was formed by covalent attachment of p-carboxyphenylamino mannose (PCAM) to gold nanoparticles (AuNPs) electrodeposited on the surface of a glassy carbon electrode (GCE). The resultant structure of PCAM was characterized and confirmed using a Fourier Transform Infrared Spectrometer (FTIR). The developed biosensor demonstrated a linear response with a logarithm of bacterial concentration (R^2^ = 0.998) in the range of 1.3 × 10 ^1^~1.3 × 10^6^ CFU·mL^−1^ with the limit of detection of 2 CFU·mL^−1^ within 60 min. The sensor did not generate any significant signals with two non-target strains, demonstrating the high selectivity of the developed biorecognition chemistry. The selectivity of the sensor and its applicability to analysis of the real samples were investigated in tap water and low-fat milk samples. Overall, the developed sensor showed to be promising for the detection of *E. coli* pathogens in water and low-fat milk due to its high sensitivity, short detection time, low cost, high specificity, and user-friendliness.

## 1. Introduction

*Escherichia coli (E. coli)* is one of the most important Gram-negative bacteria of the *Enterobacteriaceae* family. *E. coli* is one of the major pathogens for humans and animals with a contamination dose of less than 100 colonies [1]. The most common way for it to be transmitted to humans and animals is through contaminated water and food such as undercooked meat and contaminated vegetables [2]. Although the vast majority of *E. coli* serotypes are harmless and exist in the gastrointestinal tract normally, some of them such as *E. coli O157:H7*, *E. coli O167*, etc., cause infection and are pathogenic to humans and animals [2,3]. In recent decades, *E. coli* has received a lot of attention as one of the main pathogens causing severe illnesses, such as gastrointestinal diseases, bloody diarrhea, kidney failure, stillbirth, premature birth, and infection during pregnancy [4,5,6,7,8]. According to the report of the Centers for Disease Control (CDC), *E. coli* infection causes approximately 125,000 hospitalizations and 3000 deaths in the U.S. each year [2]. Therefore, early detection and prevention of the spread of this pathogen in all countries, including developed countries, is critical.

Given the importance of understanding the mechanism *of E. coli* pathogenicity, many studies have concentrated on investigating interactions between animal tissues and the bacterium. Sharon et al. reported that many strains of *E. coli* and other bacteria from the *Enterobacteriaceae* family attach to the host cell by FimH, a two-domain protein at the tip of type I fimbriae (i.e., pili) on the bacterial surface. FimH contains a mannoside-binding lectin domain having a high affinity for D-mannose (D-MAN). D-MAN is a six-carbon sugar from the aldohexose group that is abundant in the human body, especially in epithelial cells [9,10,11].

Traditional methods based on media culture and colony counting are the most common methods for identifying *E. coli*. One of the most important limitations of these methods is that they are time-consuming (2 to 5 days). New methods for rapid detection of *E. coli* bacteria, such as Polymerase Chain Reaction (PCR), Enzyme-Linked Immunosorbent Assay (ELISA), and real-time PCR, have been developed in recent decades [12]. Although these methods have high accuracy and speed, the high cost and need for specialized knowledge and complex equipment are their disadvantages [13]. Several types of biosensors such as the aptasensor [14], immunosensor [15], carbohydrate biosensors [16], and enzymatic biosensor [17] have been developed to identify pathogens, including *E. coli* bacteria, using different techniques such as electrochemical [18], colorimetric [19], optical [20], and quartz crystal microbalance (QCM) methods [21].

Among various detection methods, electrochemical detection stands out due to its simplicity, cost-effectiveness, and short response time. The Electrochemical impedance Spectroscopy (EIS) technique, in particular, is a highly sensitive method for accessing changes in electron transfer resistance at the electrode surface [22]. Wang et al. developed an aptamer-based electrochemical biosensor for the detection of *E. coli* in licorice extract with a limit of detection of 80 CFU·mL^−1^ [23]. Zhang et al. fabricated an electrochemical biosensor for *E. coli* detection using 16S rDNA as a target biomarker and oligonucleotide probes for *E. coli* detection [24]. Yang et al. reported an impedimetric biosensor based on lectin functionalized mixed self-assembled monolayer for *E. coli* detection with the limit of detection of 75 CFU·mL^−1^ [25].

This study is of a carbohydrate-based electrochemical sensor. D-MAN was immobilized on the GCE surface in a simple way, in which immobilized D-MAN on the surface of gold nanoparticle (AuNP)-modified GCE was used as a receptor for the detection of *E. coli*. Previous works reported using carbohydrate-based sensors were based on QCM, SPR, and optical techniques for *E. coli* detection. On the other hand, D-MAN is an available and cost-effective material and has advantages compared to biological elements such as aptamers or antibodies. The mechanism of bacterial detection in the proposed sensor is based on the interaction between MAN and FimH at the tip of the pili located on the outer membrane of *E. coli*. The results of this interaction were evaluated using EIS, Differential Pulse Voltammetry (DPV), and Cyclic Voltammetry (CV). The developed sensor is simple and sensitive and can detect *E. coli* within 60 min and discriminate between *E. coli* and other bacteria.

## 2. Experimental Methods

### 2.1. Materials 

All the reagents used were of analytical grade. HAuCl_4_ (Sigma-Aldrich, St. Louis, MI, USA), pure ethanol (100%) (Merck, Darmstadt, Germany), K_3_Fe(CN)_6_ and K_4_Fe(CN)_6_ (Sigma-Aldrich, St. Louis, MO, USA), Dimethyl aminopropyl-3-ethyl carbodiimide (EDC), and N-hydroxysuccinimide (NHS) were purchased from Sigma-Aldrich (St. Louis, MO, USA). Cysteamine (Cyst), 4-aminobenzoic acid (PABA), acetic acid, D-MAN, KH_2_PO_4_, K_2_HPO_4_, NaOH, and alumina powder were purchased from Merck (Darmstadt, Germany). Deionized water (DI water) was obtained from Bandar Imam Petrochemical company in Mahshahr, Iran. The microorganisms *E. coli* (PTCC 1399), *Staphylococcus epidermidis* (PTCC 1856), and *Citrobacter freundii* (PTCC 1600) were collected from a Persian Type Culture Collection (Karaj, Iran). The Phosphate Buffered Saline (PBS) solution (pH 7.4) was used to prepare different bacterial concentrations by the serial dilution method for all of the strains. R2A Agar (Scharlau, Microbiology 01-540-500, Sentmenat, Barcelona, Spain) was used for the culture and count, and Nutrient Broth (NB) (Scharlau, Microbiology 02-1440-500, Sentmenat, Barcelona, Spain) was used for the regeneration of all types of bacteria.

### 2.2. Apparatus

Electrochemical measurements were done using the Metrohm Autolab potentiostat (model PGSTATAT302N, Bijdorpplein, Barendrecht, The Netherlands). Modified GCE (Azar Electrode, Orumieh, Iran), a Ag/AgCl electrode (Azar Electrode, Orumieh, Iran), and platinum wire (Azar Electrode, Orumieh, Iran) were used as working, reference, and counter electrodes, respectively. An ultrasonic bath (Euronda—Eurosonic 4D—Via Chizzalunga, Sandrigo, Italy) was used to ensure complete cleaning of the GCE surface. Additionally, a colony counter (WTW, BZG 30, Berlin, Germany), Vortex (IKA, WORTEX GENIUS 3, Staufen im Breisgau, Germany), and a pH meter (Metrohm, Ionenstrasse, Herisau, Switzerland) were used. The morphological structure of the modified electrode surface was examined using a Field Emission Scanning Electron Microscope (FESEM) (MIRA3TESCAN-XMU-TESCAN, Brno—Kohoutovice, Czech Republic) and EDS spectroscopy (Energy-dispersive X-ray) taken on a Vega-Tescan electron microscope.

### 2.3. Synthesis of Biorecognition Element

D-MAN was modified according to the previously reported method [26]. D-MAN (0.1816 gr) was dissolved in water (5 mL), and 4-aminobenzoic acid (0.13714 gr) was solved in 5 mL acetic acid. The mannose was mixed with p-amino benzoic acid solutions and then incubated in seal tubes for 1 h at 20 °C for the amination reaction step. Then, the reduction step was carried out overnight in fume hood in an unsealed tube. As acetic-acid evaporates, p-carboxyphenylamino mannose (PCAM) single crystals were formed and used as a bioreceptor in this work. The resulting bioreceptors were stored at −20 °C under light- and moisture-blocking conditions. The name and identity of the PCAM were predicted using Chem Draw and confirmed with FTIR spectra.

### 2.4. Sensor Fabrication

**Scheme 1:**
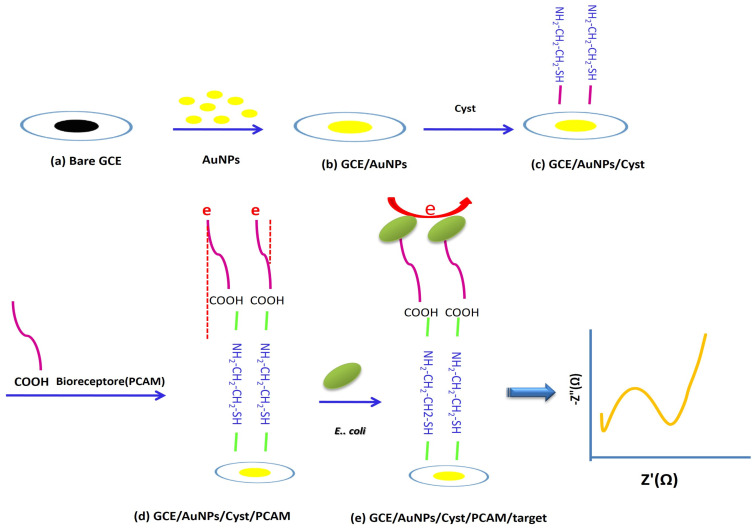
Schematic diagram of label-free carbohydrate-based electrochemical sensor for the detection of *E.coli* cells. (**a**) GCE bare, (**b**) electrodeposition AuNPs and formation GCE /AuNPs, (**c**) electrode incubation with Cyst and formation of GCE /AuNPs/Cyst, (**d**) stabilization of PCAM on the surface of the electrode and formation of GCE /AuNPs/Cyst/PCAM, (**e**) detection of E. coli bacteria by modified electrode (GCE /AuNPs/Cyst/PCAM).

The GCEs were polished with alumina powder (0.3, 0.1, and 0.05 μm) and then carefully washed with DI water. To ensure a well-cleaned surface, the GCEs were sonicated in an ultrasonic bath containing DI water and ethanol in a 1: 1 ratio for 3 min. They were then rinsed again with DI water and dried at room temperature. AuNPs were deposited on the surface of the GCEs by electrochemical deposition. AuNPs on the surface of a GCE increase the effective electrode surface and the sensitivity of electrochemical analyses and provide a unique platform for increased loading of receptors. A GCE was immersed in a solution containing 5 mmol.L^−1^ HAuCl_4_ and 0.5 mol.L^−1^ H_2_SO_4_, and the potential of −0.2 V was applied for 200 s [27]. The AuNPs/GCE electrode was immersed in 0.001 M Cyst for 17 h. Cyst was used as a linker because it could stabilize the activated PCAM (bioreceptor) on the surface of AuNPs/GCE. Then, the electrode was rinsed with DI water to remove the unbound Cyst on the electrode surface. PCAM was dissolved in 1 mL acetic acid and DI water (1: 1) for 20 min. After that, the electrode was activated using 0.0035 mg.mL^−1^ of EDC and 0.0028 mg.mL^−1^ of NHS for 1 h. In the next step, the activated Cyst/AuNPs/GCE electrode was immersed in the PCAM solution for 3 h, and the electrode surface was thoroughly washed with DI water and PBS (pH 7.4) to remove the unbound bioreceptor and avoid false-positive responses. Finally, the electrode was stabilized in PBS (pH 7.4) for 24 h. All preparation stages of the proposed biosensor were monitored by EIS, DPV, and Cyclic Voltammetry (CV) methods. Resistance changes (R_et_) were measured at all fabrication steps of the biosensor to evaluate the performance of the proposed biosensor. All measurements were repeated at least three times. The overall process is shown in Scheme 1.

### 2.5. Electroanalytical Measurements

All electrochemical measurements were conducted in a standard three-electrode system in a glass cell containing 18 mL of 0.1 mol.L^−1^ KCl and 5.0 mmol.L^−1^ K_3_[Fe(CN)_6_]/K_4_[Fe(CN)_6_] (1:1) as a redox probe. For bacteria detection, the modified electrodes were incubated at concentrations of 1.3 × 10 to 1.3 × 10^6^ CFU·mL^−1^ *E. coli* for 1 h. After incubation with 1 mL of bacterial sample, the electrode was thoroughly rinsed with DI water to wash bacteria that were not attached to the electrode surface or had poor binding to prevent a false positive response.

EIS measurement, which is an accurate and sensitive method to evaluate electron transfer changes at the electrode surface, was performed to evaluate R_et_ changes at the electrode surface for all steps of fabrication and detection of *E. coli* bacteria and obtain the calibration curve. CV was performed to further evaluate the electrochemical performance of the proposed sensor and also to further confirm the results obtained from EIS. EIS measurements were performed in the frequency range of 0.1 Hz to 100 kHz, the potential of 0.24 V, and the amplitude of 0.01 V. CV measurements were done within the potential range of −0.4 to 0.8 V at a scan rate of 10 mV.s^−1^. All preparation stages of the proposed biosensor were monitored by EIS, DPV, and CV methods. R_et_ was measured at all fabrication steps of the biosensor to evaluate the performance of the proposed biosensor. All measurements were repeated at least three times. The overall process is shown in Scheme 1.

### 2.6. Real Sample Preparation

To evaluate the performance of the proposed biosensor, tap water and low-fat milk were prepared as real samples. Samples were spiked with *E. coli* to obtain the final concentrations of 10, 10^4^, 10^5^, and 10^6^ CFU·mL^−1^. EIS was performed for the samples, and according to the calibration plot, the recovery percentage and RSD% were determined. It is also important to mention that we used a sample of low-fat milk purchased from the supermarket without special preparation or screening.

### 2.7. Bacterial Culture and Counting Methods

*E. coli* (PTCC 1399) as the target bacterium and *Staphylococcus epidermidis* (PTCC 1856) and *Citrobacter freundi* (PTCC 1600) as the non-target species were cultivated in NB at 37 °C for 20 h. Bacteria cells were harvested by centrifugation for 5 min at 3500 rpm. The cells were then re-suspended in PBS (0.1 mol.L^−1^, pH 7.4) and washed by centrifugation (three times). The resulting solution in PBS (0.1 mol.L^−1^, pH 7.4) was used as a stock solution for the detection of *E. coli* and other bacterial species. The serial dilution method in PBS (0.1 mol.L^−1^, pH 7.4) was used to prepare different concentrations of bacteria from the stock solution. Bacterial populations were counted on the R2A agar plate before use by the conventional colony counting method.

### 2.8. Sensor Concept and Design of Modified Mannose

**Scheme 2:**
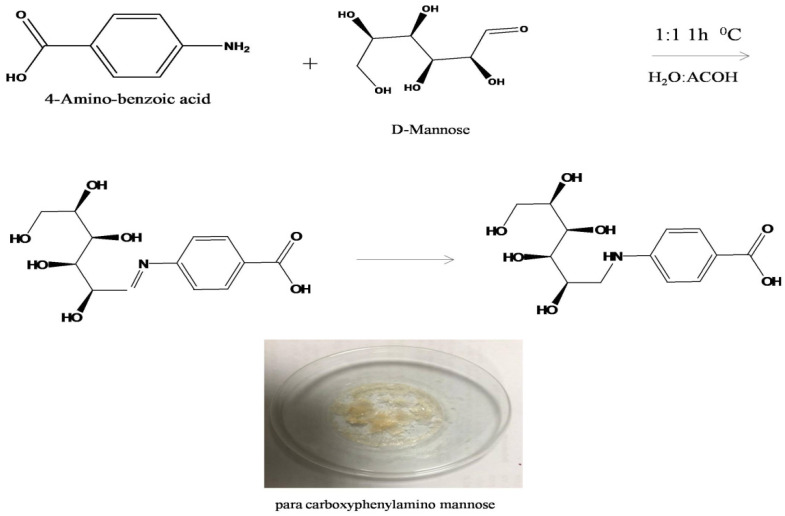
The steps of para carboxyphenylamino mannose synthesis.

The concept of the sensor is based on the interaction between type I fimbriae in the outer membrane of *E. coli* bacteria and mannose ligands (PCAM) fixed on the surface, which has strong binding and high selectivity to this bacterium compared to other bacteria. The steps of the synthesis of PCAM using Chem Draw software are shown in Scheme 2. This method surface modification method is very cost-effective and can be applied to other receptors such as aptamers and antibodies.

### 2.9. Material Selection

In this study, AuNPs were used as a material to improve the surface of GCE and D-MAN as capture prob. AuNPs are widely used in many fields for their unique optical and physical properties. AuNPs with unique properties, such as a high specific surface area, can provide a wide surface for coating carbohydrate ligands or other ligands. Further, these nanoparticles are highly conductive, which improves the transfer of electron current on the surface of the electrode. AuNPs can be conjugated with many functionalizing agents, such as polymers, ligands, dendrimers, drugs, DNA, RNA, and proteins. Another feature of these nanoparticles is the ability to form strong covalent bonds with thiol groups that provide the possible formation of SAM on the surface with other materials.

D-MAN is a carbohydrate that can be chemically modified to contain functional groups such as carboxyl or thiol and create Self-Assembled Monolayers (SAMs) on the electrode surface through reaction with other groups. This carbohydrate has an affinity attached to the adhesion FimH on the tip of type I fimbriae in the outer membrane of Escherichia coli bacteria that was used as a receptor in this work.

## 3. Results and Discussion

### 3.1. Surface Characterization

**Figure 1:**
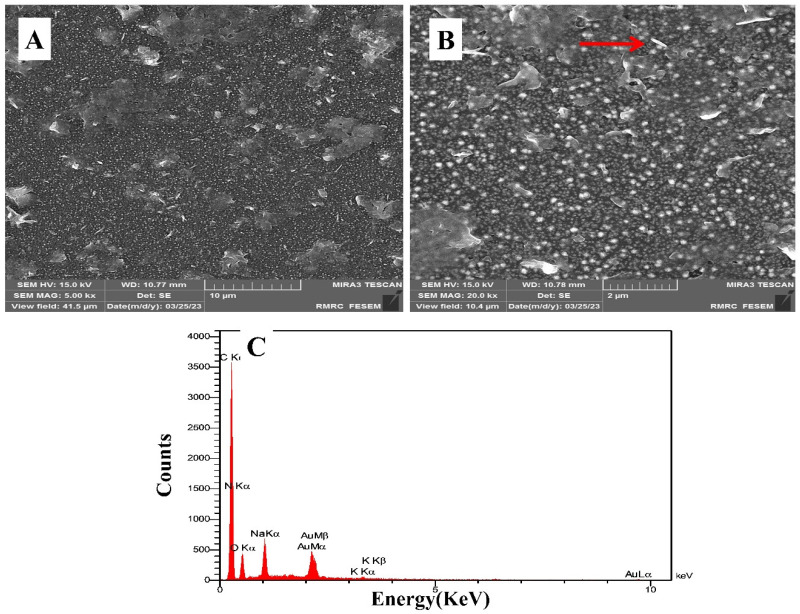
FESEM images of the electrodeposited gold nanoparticles and *E. coli* captured by PCAM on the modified GCE surface, (**A**) 10 μm scale and (**B**) 2 μm scale; the red arrow points to *E. coli*. (**C**) EDS of the modified surface of GCE.

The morphologies of the AuNPs electrodeposited on the surface of GCE, under the deposition conditions mentioned in Section 2.4, were characterized with FESEM images. The FESEM micrograph in Figure 1A indicates that an appropriate layer of AuNPs with a nanoparticle size is formed on the surface of GCE; hence, as seen, the FESEM images indicate a satisfactory uniform coverage of AuNPs on the surface of GCE. Moreover, to visualize the capture of bacteria by the PCAM-coated electrode, we used FESEM to confirm *E. coli* on the sensor surface. The FESEM micrograph in Figure 1B indicates the captured *E. coli* on the electrode surface, which is shown by the red arrow. Furthermore, it is established that Energy-dispersive X-ray Spectroscopy (EDS) penetrates much more deeply and hence gives the composition of bulk materials. As shown in Figure 1C, the EDS spectrum confirms the presence of Au on the GCE surface, and the peaks of C, N, and O in the EDS spectrum prove the stabilization of mannose on the surface of the electrode.

### 3.2. FTIR Characterizations

**Figure 2:**
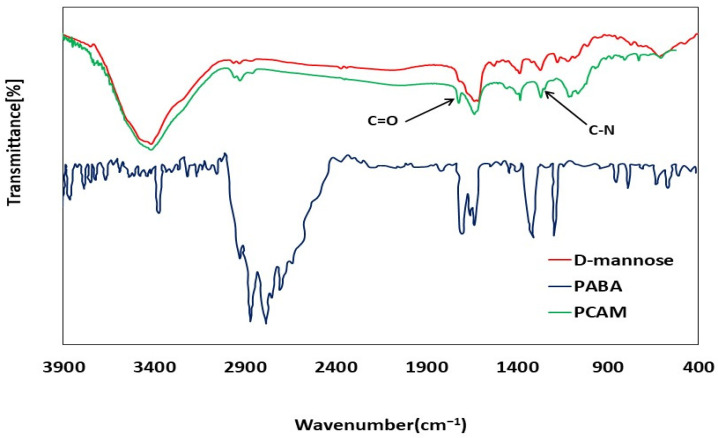
FTIR mannose analysis and mannose derivation (PCAM).

The FTIR analysis was performed to evaluate the synthesis of the PCAM receptor. The PCAM receptor was obtained from the reaction between two substances, para-aminobenzoic acid and D-MAN. According to the FTIR spectrum of para-aminobenzoic acid, a peak corresponding to C=O at the wavenumber of 1710 cm^−1^ is expected to be seen in the FTIR spectrum of the PCAM receptor, compared to pure MAN (Figure 2). In the FTIR of the PCAM receptor, this peak was seen at 1710 cm^−1^, indicating that the PCAM was successfully synthesized.

Additionally, the peak observed in the wavenumber range of 1342–1266 cm^−1^ is related to C-N, which shows the presence of the aromatic amine of synthesized PCAM. However, it is not seen in pure MAN, which indicates the reaction between para-aminobenzoic acid and MAN.

### 3.3. Electrochemical Characterization

**Figure 3:**
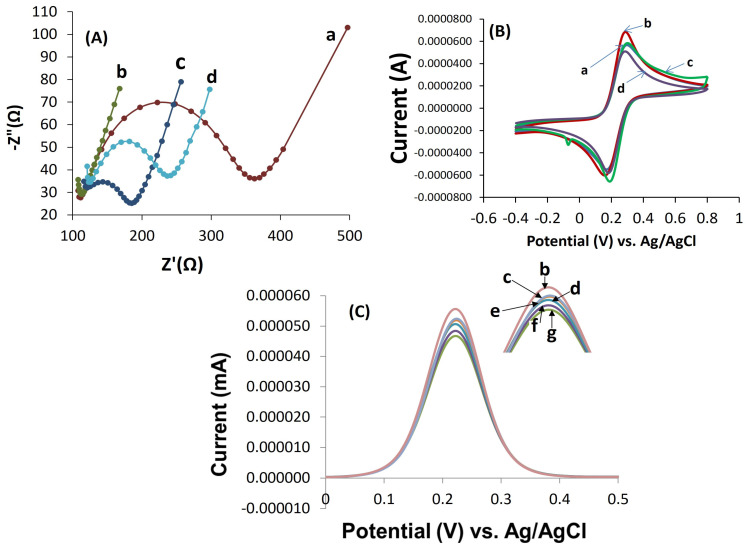
(**A**) EIS Nyquist diagrams (−Z″ vs. Z′) of modified electrodes: (a) bare GCE, (b) AuNPs/GCE, (c) PCAM/Cyst/AuNPs/GCE, (d) *E. coli*/PCAM/Cyst/AuNPs/GCE. (**B**) CV and (**C**) DPV curves of the proposed nano-electrochemical biosensor after incubating with different concentrations of *E. coli* 1.3 × 10^1^–1.3 × 10^6^ CFU·mL^−1^ (from top to bottom; e to g) in [Fe(CN)_6_]^3−/4−^ as electrolyte solution.

The results of electrochemical measurements of EIS in each stage of the sensor fabrication in the K_3_[Fe(CN)_6_]/K_4_[Fe(CN)_6_] solution as a redox probe are shown in Figure 2A. The R_et_ of bare GCE was obtained to be 250 Ω. After electrochemical deposition of AuNPs, R_et_ was greatly reduced, and the resulting spectrum became almost linear, indicating that the charge transfer resistance was significantly reduced due to electrode coverage with high-conductive AuNPs. When the AuNPs/GCE electrode was immersed in the Cyst solution to stabilize PCAM (bioreceptor) on the surface of AuNPs/GCE, the diameter of the semicircle in the Nyquist diagram was increased, which indicates an enhanced surface resistance to 60 Ω and successful stabilization of PCAM on the electrode surface (Figure 3A).

According to Figure 3A, EIS measurements with increasing concentrations of *E. coli* showed an increase in R_et_ up to 120 Ω, which confirmed the detection of bacteria by the proposed biosensor. To further ensure the performance of the designed biosensor, DPV and CV measurements were performed simultaneously for different bacterial concentrations. CV was performed for further characterization of electrode surface modifications. The cyclic voltammograms were recorded within the potential range of −0.4 to 0.8 V. The recorded cyclic voltammograms for different steps of the modified electrode showed the conductivity of bare GCE (a) so that the conductivity of surface electrode extremely increased after electrodeposition of AuNPs on the GCE surface (b). However, after the immobilizing of the receptor (PCAM) (c) and also exposing the sensor to *E. coli* bacteria (d), the conductivity of the sensor was decreased. These measurements are in good agreement with the results obtained from EIS; both sets confirm the successful fabrication and operation of the biosensor (Figure 3B). DPV results showed the current response was decreased by increasing the concentration of *E. coli* (Figure 3C). In contrast to CV measurements, increasing the concentration of *E. coli* decreased the redox current at the surface. The results of all three measurement methods confirmed each other.

### 3.4. Optimization of Incubation Time

**Figure 4:**
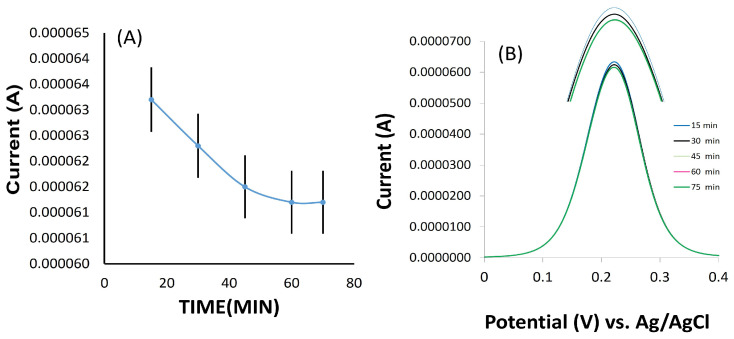
(**A**) Responses of electrochemical biosensor *E. Coli* (1.3 ×10^5^ CFU·mL^−1^) in [Fe(CN)_6_]^3−/4−^ as an electrolyte solution as a function of incubation time (from top to bottom 0–60 min); (**A**) the plot of peak current versus incubation time, (**B**) DPV responses in different incubation times.

One of the advantages of biosensors over conventional bacteria detection methods such as culture medium is their high speed in detecting bacteria. In order to prove this and also to evaluate the proposed biosensor, the optimal incubation time to attach bacteria to the electrode surface was calculated. The modified electrode was incubated in an *E. coli* solution with 10^5^ CFU·mL^−1^ concentration. According to Figure 4, the electrochemical response of the biosensor was increased until it was stable after 1 h incubation. Therefore, 1 h was chosen as the optimal incubation time for *E. coli* in all experimental steps. This step of the experiment was repeated at least three times. The shorter incubation time means that the identification of bacteria by the biosensor will be conducted in a shorter period of time, which is one of the most important advantages of biosensors over conventional methods such as the culture medium.

### 3.5. Biosensor Calibration Curve and Limit of Detection

**Figure 5:**
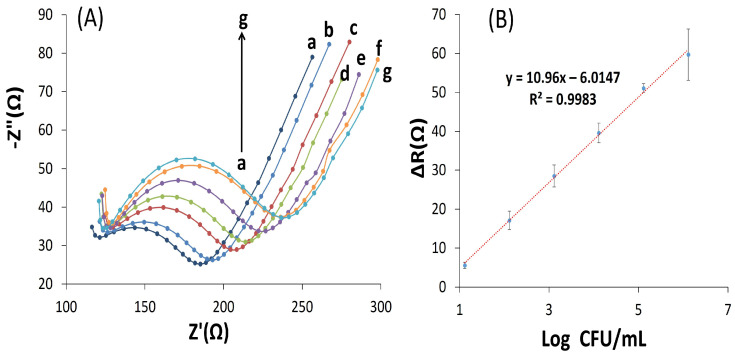
(**A**) EIS was performed in a frequency range of 0.1 Hz to 100 kHz, at a potential of 0.24 V, and amplitude of 0.01 V at different concentration of *E. coli,* including (a): PCAM, (b): 1.3 × 10^1^, (c): 1.3 × 10^2^, (d): 1.3 × 10^3^, (e): 1.3 × 10^4^, (f): 1.3 × 10^5^, (g): 1.3 × 10^6^ CFU·mL^−1^_._ (**B**) Calibration curve obtained for ΔR versus Log of *E. coli* concentration in [Fe(CN)_6_]^−3/−4^.

The calibration curve was obtained using EIS at different concentrations of *E. coli* bacteria. Changes in the EIS and DPV responses of the fabricated biosensor were evaluated at 1.3 × 10^1^–1.3 × 10^6^ CFU·mL^−1^ concentrations of *E. coli*. According to the results of the EIS measurement, R_et_ values were obtained for each concentration, and ΔR_et_ was calculated from the obtained values. Then, ΔR_et_ versus logarithmic concentration of *E. coli* was plotted in the range of 1.3 × 10^1^–10 × 10^6^ CFU·mL^−1^. According to Figure 5B, the calibration curve shows a linear range with the correlation equation y = 10.96x − 6.014 (R^2^ = 0.998). The limit of detection (LOD) of the biosensor was determined to be 2 CFU·mL^−1^ (three times the standard detection of the blank experiment: incubation in the absence of *E. coli*) based on EIS measurements before and after the addition of *E. coli* bacteria.

Compared to the previous works conducted with carbohydrate receptors to identify pathogenic *E. coli* bacteria, the results of this study showed a high linear range at 1.3 × 10^1^–10 × 10^6^ CFU·mL^−1^. Further, the previous works have been conducted with more expensive and complicated methods such as QCM or SPR. Although they obtained good accuracy and sensitivity, these methods were expensive and complicated. In addition, the use of intermediary materials caused more complexity and higher cost. In this research, a simple electrochemical method and an inexpensive electrode GCE as a working electrode and support for MAN were used. *E. coli* bacteria were accurately and sensitively identified with 2 CFU·mL^−1^ with a great linear range, which shows the excellent performance of the proposed biosensor compared to previous works (Table 1). The proposed biosensor was also evaluated after 23 days of storage in phosphate buffer, and no decrease in the efficiency of bacteria identification was observed by the proposed biosensor, which indicates the excellent stability of the biosensor.

### 3.6. Selectivity of the Biosensor

**Figure 6:**
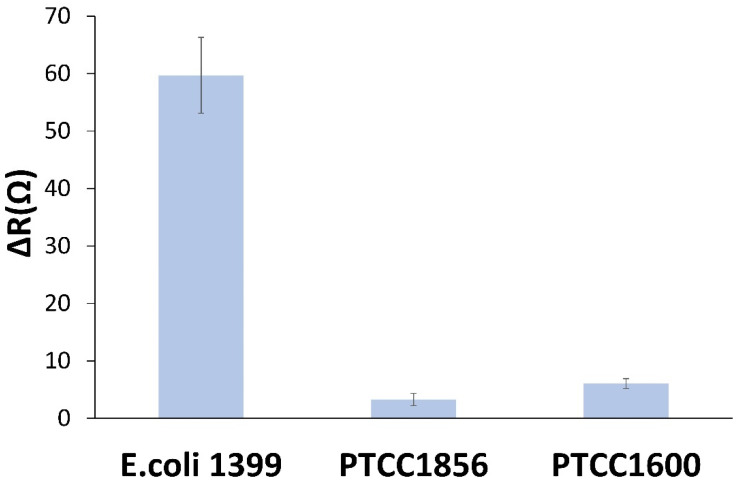
Selectivity experiments using 10^6^ CFU·mL^−1^ solutions of *E. coli*, *Staphylococcus epidermidis* (PTCC 1856), and *Citrobacterfreundii* (PTCC 1600).

One of the most important parameters for evaluating the performance of a biosensor is the ability of the sensor to distinguish between target and non-target samples. The sensor specificity was evaluated in *E. coli* samples of target and two non-target strains, including *Staphylococcus epidermidis* (PTCC 1856) and *Citrobacter freundii* (PTCC 1600), which tend to bind to MAN. The biosensor was incubated in the 10^6^ CFU·mL^−1^ solution of each bacterium for 1 h, and EIS measurements were performed for all bacterial strains. As shown in Figure 6, the impedimetric biosensor did not show a significant response to the two non-target bacterial species. Additionally, the results of evaluating the selectivity of the sensor and the selectivity coefficient are shown in Table S1.

### 3.7. Real Sample Measurement

To evaluate the performance of the proposed biosensor in real samples, owing to the importance of water and food safety, the biosensor was tested in tap water and low-fat milk samples. Samples were spiked at target concentrations of 10, 10^2^, 10^3^, 10^4^, 10^5^, and 10^6^ CFU·mL^−1^. The biosensors were then incubated in the spiked samples. The results of biosensor recovery for water samples are reported in Table 2. As shown in Table 3, the obtained recoveries for lower concentrations of bacteria are more acceptable.

**Figure 7:**
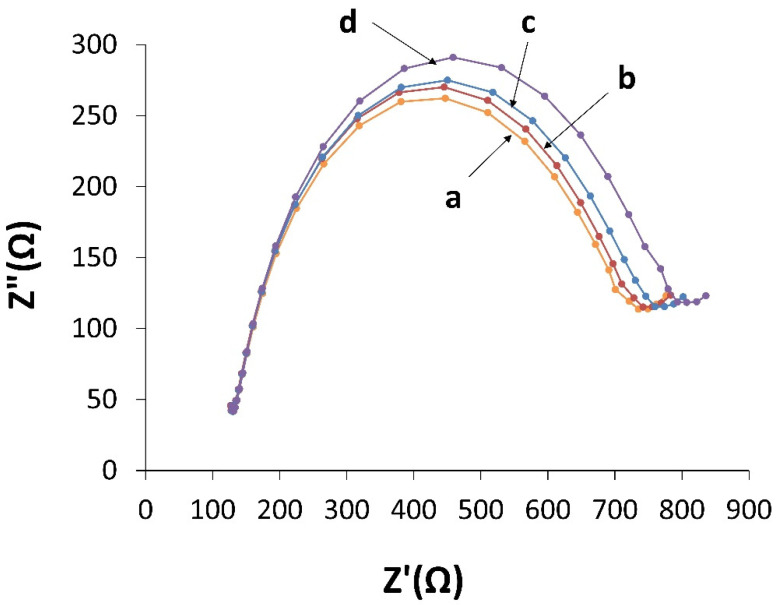
EIS curves for the proposed biosensor in a solution containing [Fe(CN)_6_]^−3/−4^ after incubation with milk sample in a frequency range of 0.1 Hz to 100 kHz, at a potential of 0.24 V, and an amplitude of 0.01 V at different concentrations of *E. coli,* including (a): 1.3 × 10^1^, (b): 1.3 × 10^2^, (c): 1.3 × 10^3^, and (d): 1.3 × 10^5^ CFU·mL^−1^.

**Figure 8:**
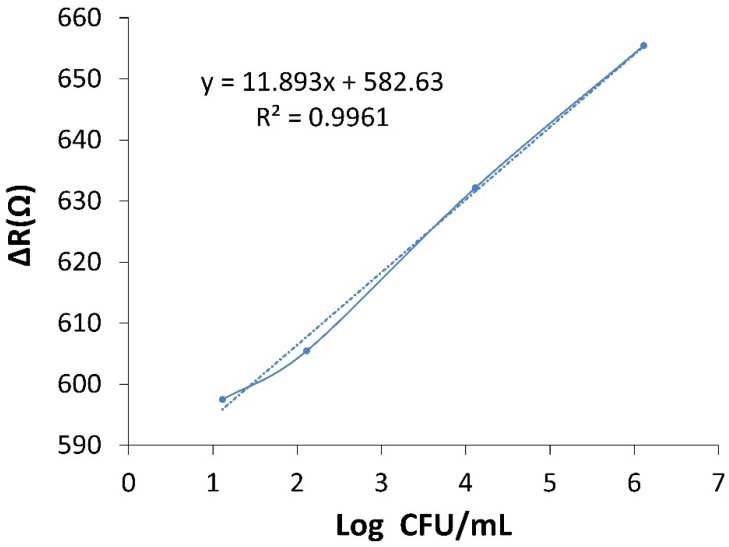
The plot ΔR versus Log of *E. coli* concentration.

For the milk sample, we used the low-fat milk sample purchased from a supermarket without pre-preparation or screening. The graph obtained from the impedance measurement based on the logarithm of the bacterial concentration against the resistance changes for the final concentrations of 10^1^, 10^2^, 10^3^, and 10^6^ was plotted (Figure 7). The slope of the resulting line shows the change in resistance caused by the change in the number of bacteria (Figure 8). From the comparison of the slope of the resulting line of 11.893 with the slope of the curve calibration graph line of 10.96, the amount of deviation from the standard state of the biosensor was calculated to be 7.844% (Table 3). On the other hand, the width from the origin of the graph depends on the properties of the fluid. As indicated in the graph, the width from the origin in the graph of the valve is 582.63 Ω, which is larger than the width from the origin in the graph of the sensor calibration curve (6.014 Ω), which can be due to the properties of proteins and impurities in milk.

## 4. Conclusions

A label-free carbohydrate-based biosensor was proposed for the sensitive and accurate identification of *E. coli* bacteria using D-MAN and AuNPs. FE-SEM, FTIR, EDS, EIS, and CV were performed to characterize and confirm the electrode surface fabrication. The fabricated biosensor with advantages such as excellent low LOD (2 CFU·mL^−1^) was able to identify this pathogenic bacterium in a very short time of 1 h and did not show a significant response to the non-target bacterial species, indicating the high selectivity of the proposed biosensor. The manufactured biosensor was successfully used in a tap water sample and low-fat milk with acceptable recoveries, which shows the prospect for the fast and accurate on-site assay of environmental and food products. Another advantage of this biosensor is the use of a very inexpensive and available carbohydrate bioreceptor compared to other biosensors such as antibodies and aptamers. Additionally, the proposed method in this work can be used for other bacteria by changing the bioreceptor.

## Figures and Tables

**Table 1:** Comparison of the fabricated biosensor with other works for the detection of *E. coli*.

Method	Linear Dynamic Range	LOD	Reference
Electrochemical and quartz crystal microbalance polythiophene interface biosensor using Concanavalin A	1.0 × 10^2^–5.0 × 10^3^ cells. mL^−1^	25 cell.mL^−1^ for electrochemical sensor and 50 cells.mL^−1^ for QCM sensor	[15]
Lectin-based impedimetric biosensor	10^2^–10^5^ cells. mL^−1^	75 cells. mL^−1^	[25]
Carbohydrate-based label-free biosensor for detection of *E. coli ORN 178*	10^2^–10^3^ CFU·mL^−1^	10^2^ CFU·mL^−1^	[28]
Portable nanofiber-light addressable potentiometric sensor for rapid detection of *E. coli*	-	10^2^ CFU·mL^−1^	[29]
QCM Biosensor using carbohydrate and lectin	7.5 × 10^2^ to 7.5 × 10^7^ cells. mL^−1^	7.5 × 10^2^ cells.mL^−1^	[30]
Electrochemical carbohydrate-based biosensor	1.3–1.3 × 10^6^ CFU·mL^−1^	2 CFU·mL^−1^	This work

**Table 2:** Detection of *E. coli* in water samples.

Sample	Concentration (CFU·mL^−1^)	ΔR in Real Sample	ΔR in Calibration Curve	Recovery (%)	RSD (%)
Tap Water	10^4^	43.791	45.099	97.10	3.84
	10^5^	71.491	67.231	106.34	2.37
	10^6^	109.891	92.221	119.16	4.13

**Table 3:** Deviation of calibration curve.

Slope in Milk	Slope in Calibration Curve	Deviation from the Calibration Curve
11.893	10.96	7.844

## Data Availability

The data that support the findings of this study are available from the corresponding author upon reasonable request.

## Supplementary Materials

The following supporting information can be downloaded at: https://www.mdpi.com/article/10.3390/bios13060619/s1, Table S1: The results of sensor selectivity evaluation.

## References

[1] Doyle M.P., Caballero B. (2013). Food Safety: Bacterial Contamination. Encyclopedia of Human Nutrition.

[2] Mead P.S., Slutsker L., Dietz V., McCaig L.F., Bresee J.S., Shapiro C., Griffin P.M., Tauxe R.V. (1999). Food-related illness and death in the United States. Emerg. Infect. Dis..

[3] Thomas L.V., Rowe B., McConnell M.M. (1987). In strains of *Escherichia coli* O167 a single plasmid encodes for the coli surface antigens CS5 and CS6 of putative colonization factor PCF8775, heat-stable enterotoxin, and colicin Ia. Infect. Immun..

[4] Krohn M.A., Thwin S.S., Rabe L.K., Brown Z., Hillier S.L. (1997). Vaginal colonization by *Escherichia coli* as a risk factor for very low birth weight delivery and other perinatal complications. J. Infect. Dis..

[5] Banatvala N., Griffin P.M., Greene K.D., Barrett T.J., Bibb W.F., Green J.H., Wells J.G. (2001). The United States National Prospective Hemolytic Uremic Syndrome Study: Microbiologic, Serologic, Clinical, and Epidemiologic Findings. J. Infect. Dis..

[6] Li S., Konoval H.M., Marecek S., Lathrop A.A., Feng S., Pokharel S. (2023). Control of *Escherichia coli* O157: H7 using lytic bacteriophage and lactic acid on marinated and tenderized raw pork loins. Meat Sci..

[7] Dester E., Kao K., Alocilja E.C. (2022). Detection of unamplified *E. coli* O157 DNA extracted from large food samples using a gold nanoparticle colorimetric biosensor. Biosensors.

[8] Chan M.Y., Smith M.A. (2018). Infections in pregnancy. Compr. Toxicol..

[9] Mayer K., Eris D., Schwardt O., Sager C.P., Rabbani S., Kleeb S., Ernst B. (2017). Urinary tract infection: Which conformation of the bacterial lectin FimH is therapeutically relevant?. J. Med. Chem..

[10] Firon N., Ofek I., Sharon N. (1984). Carbohydrate-binding sites of the mannose-specific fimbrial lectins of enterobacteria. Infect. Immun..

[11] McClure E.M., Goldenberg R.L. (2009). Infection and Stillbirth.

[12] Singh A., Poshtiban S., Evoy S. (2013). Recent advances in bacteriophage based biosensors for food-borne pathogen detection. Sensors.

[13] Spagnolo S., De La Franier B., Davoudian K., Hianik T., Thompson M. (2022). Detection of *E. coli* bacteria in milk by an acoustic wave aptasensor with an anti-fouling coating. Sensors.

[14] Sobhan A., Jia F., Kelso L.C., Biswas S.K., Muthukumarappan K., Cao C., Wei L., Li Y. (2022). A Novel Activated Biochar-Based Immunosensor for Rapid Detection of *E. coli* O157: H7. Biosensors.

[15] Ma F., Rehman A., Liu H., Zhang J., Zhu S., Zeng X. (2015). Glycosylation of quinone-fused polythiophene for reagentless and label-free detection of *E. coli*. Anal. Chem..

[16] Kaur J., Choudhary S., Chaudhari R., Jayant R.D., Joshi A., Pal K., Kraatz H.-B., Khasnobish A., Bag S., Banerjee I., Kuruganti U. (2019). 9—Enzyme-based biosensors. Bioelectronics and Medical Devices.

[17] Yao W., Shi J., Ling J., Guo Y., Ding C., Ding Y. (2020). SiC-functionalized fluorescent aptasensor for determination of Proteus mirabilis. Microchim. Acta.

[18] Xiao S., Yang X., Wu J., Liu Q., Li D., Huang S., Xie H., Yu Z., Gan N. (2022). Reusable electrochemical biosensing platform based on egg yolk antibody-labeled magnetic covalent organic framework for on-site detection of *Escherichia coli* in foods. Sens. Actuators B Chem..

[19] Zhang G., Hu H., Deng S., Xiao X., Xiong Y., Peng J., Lai W. (2023). An integrated colorimetric and photothermal lateral flow immunoassay based on bimetallic Ag–Au urchin-like hollow structures for the sensitive detection of *E. coli* O157: H7. Biosens. Bioelectron..

[20] Gupta A., Garg M., Singh S., Deep A., Sharma A.L. (2020). Highly sensitive optical detection of *Escherichia coli* using terbium-based metal–organic framework. ACS Appl. Mater. Interfaces.

[21] Ngo V.K.T., Nguyen D.G., Nguyen H.P.U., Nguyen T.K.M., Huynh T.P., Lam Q.V., Huynh T.D., Truong T.N.L. (2014). Quartz crystal microbalance (QCM) as biosensor for the detecting of *Escherichia coli* O157: H7. Adv. Nat. Sci. Nanosci. Nanotechnol..

[22] Kaur K., Chelangat W., Druzhinin S.I., Karuri N.W., Müller M., Schönherr H. (2021). Quantitative *E. coli* enzyme detection in reporter hydrogel-coated paper using a smartphone camera. Biosensors.

[23] Wang H., Zhao Y., Bie S., Suo T., Jia G., Liu B., Ye R., Li Z. (2019). Development of an electrochemical biosensor for rapid and effective detection of pathogenic *Escherichia coli* in licorice extract. Appl. Sci..

[24] Zhang J., Wang J., Zhang X., He F. (2018). Rapid detection of *Escherichia coli* based on 16S rDNA nanogap network electrochemical biosensor. Biosens. Bioelectron..

[25] Yang H., Zhou H., Hao H., Gong Q., Nie K. (2016). Detection of *Escherichia coli* with a label-free impedimetric biosensor based on lectin functionalized mixed self-assembled monolayer. Sens. Actuators B Chem..

[26] Yazgan I., Noah N.M., Toure O., Zhang S., Sadik O.A. (2014). Biosensor for selective detection of *E. coli* in spinach using the strong affinity of derivatized mannose with fimbrial lectin. Biosens. Bioelectron..

[27] Roushani M., Shahdost-Fard F. (2015). Fabrication of an ultrasensitive ibuprofen nanoaptasensor based on covalent attachment of aptamer to electrochemically deposited gold-nanoparticles on glassy carbon electrode. Talanta.

[28] Guo X., Kulkarni A., Doepke A., Halsall H.B., Iyer S., Heineman W.R. (2012). Carbohydrate-based label-free detection of *Escherichia coli* ORN 178 using electrochemical impedance spectroscopy. Anal. Chem..

[29] Shaibani P.M., Etayash H., Jiang K., Sohrabi A., Hassanpourfard M., Naicker S., Sadrzadeh M., Thundat T. (2018). Portable nanofiber-light addressable potentiometric sensor for rapid *Escherichia coli* detection in orange juice. ACS Sens..

[30] Shen Z., Huang M., Xiao C., Zhang Y., Zeng X., Wang P.G. (2007). Nonlabeled quartz crystal microbalance biosensor for bacterial detection using carbohydrate and lectin recognitions. Anal. Chem..

