# Supplementary material for: A Label-Free Carbohydrate-Based Electrochemical Sensor to Detect Escherichia coli Pathogenic Bacteria Using D-mannose on a Glassy Carbon Electrode
Source: Biosensors (Basel). 2023 Jun 5;13(6):619. doi: 10.3390/bios13060619 (PMC10296727; doi:10.3390/bios13060619)
Supplement: Supplementary file 1 [file biosensors-13-00619-s001.zip › biosensors-2380800-supplementary.pdf]

Supplementary Material

Article

A Label-Free Carbohydrate-Based Electrochemical Sensor to Detect *Escherichia coli* Pathogenic Bacteria Using D-mannose on a Glassy Carbon Electrode

Sakineh Hargol Zadeh [1], Soheila Kashanian [1,2,*] and Maryam Nazari [1]

[1] Faculty of Chemistry, Applied Chemistry Department, Razi University, Kermanshah 6714414971, Iran
[2] Nanobiotechnology Department, Faculty of Innovative Science and Technology, Razi University, Kermanshah 6714414971, Iran
* Correspondence: kashanian_s@yahoo.com

Table S1. The results of sensor selectivity evaluation.

Bacteria	Number of Bacteria (CFU.mL^{-1})	Concentration (CFU.mL^{-1})	ΔR (Average) (Ω)	STDV (Average) (%)	Selectivity Coefficient (%)
E.coli	1300000	10^6	59.70	6.58	86.48
PTCC1856	1170000	10^6	3.28	1.08	4.75
PTCC1600	1560000	10^6	6.05	0.88	8.78
